# Apelin Serum Level in Egyptian Patients with Chronic Hepatitis C

**DOI:** 10.1155/2011/703031

**Published:** 2011-10-04

**Authors:** Hala O. El-Mesallamy, Nadia M. Hamdy, Hanan H. Rizk, Abdel-Rahman El-Zayadi

**Affiliations:** ^1^Biochemistry Department, Faculty of Pharmacy, Ain Shams University, Abassia, Cairo 11566, Egypt; ^2^Tropical Medicine Department, Faculty of Medicine, Ain Shams University, Cairo 11566, Egypt

## Abstract

*Objective*. Highlighting the apelin system would present a new therapeutic target for liver disease. Apelin; endogenous ligand for the orphan receptor APJ, was recently suggested to be associated with fibrosis progression and cirrhosis in addition to insulin resistance (IR) and inflammation. The present study was conducted to evaluate blood apelin level changes among 73 chronic hepatitis C (CHC) Egyptian patients and if associated with body mass index (BMI), IR, and tumor necrosis factor-alpha (TNF-**α**). Serum apelin levels were significantly higher in patients with CHC with median value (3.25) when compared with controls (1.11), at *P* < 0.0001, with significant apelin variations among asymptomatic carriers (ASC), fibrosis, and cirrhosis patients, and also among obese and nonobese patients. Multiple regression analysis depicted that BMI, triglycerides, and total cholesterol were independent correlation factors to apelin levels, whereas TNF-**α** was found to be significantly negatively correlated to adjusted apelin in CHC patients (*r* = −0.5944, *P* < 0.0001). IR was positively correlated to adjusted apelin in CHC patients (*r* = 0.2663, *P* < 0.05). *Conclusion*. Apelin level varies among stages of CHC, which may contribute to fibrosis progression. In addition, obesity and IR could act as comorbid factors affecting apelin level in patients with CHC.

## 1. Introduction

The receptor APJ remained orphan until 1998, when Tatemoto and his coworkers isolated its endogenous ligand from bovine stomach extract. They isolated a 36-amino-acid peptide which was named apelin (from APJ endogenous ligand) [1]. Apelin exists in at least three forms, consisting of 13, 17, or 36 amino acids, all originating from a common 77-amino-acid precursor [2]. Apelin has been shown to be involved in vessel formation, where it exerts a pro-angiogenic role [3, 4], and in the regulation of cardiovascular function, by reducing arterial blood pressure, via stimulation of nitric oxide-mediated vasorelaxation [5, 6]. Moreover, apelin has recently been added to the family of adipokines [7, 8], which are adipocytokines mainly derived from adipose tissue as well as endothelial cells (ECs) in various parts of the body [2].

Hepatitis C virus (HCV) has been recognized as a major cause of chronic liver disease(s) (CLD) in Egypt [9]. The emerging role of apelin in CLD is complex, as described in a recent report linking apelin to the initiation and maintenance of the inflammatory and fibrogenic processes occurring in the fibrotic liver [10], as well as to the vascular and haemodynamic abnormalities in cirrhosis and its complications [11, 12]. However, clinical data demonstrating the role of apelin in CLD is limited, as depicted by Bertolani and Marra [13].

Although barely detectable in normal liver, production of TNF-*α* from T-cells occurs at the early onset in many types of liver injury and has been related to fibrosis progression [14]. Moreover, adipokines as leptin, resistin, visfatin, and adiponectin are recently considered as regulators of liver fibrogenesis and may explain why obesity influences fibrosis progression [15]. 

 Hepatic insulin signaling is markedly impaired in HCV patients; in addition, down regulation of hepatic insulin mediators is associated with enhanced hepatocyte apoptosis and fibrogenesis [16]. Currently some evidence supports a relationship between insulin resistance (IR) and hepatitis C on one hand, playing role in progression of liver disease [17–20], and between IR and apelin level on the other hand in cases of obesity [7]. 

Therefore, highlighting the apelin system would present a new therapeutic target for liver disease. Thus, this study aims to address if there is relation between apelin serum levels and liver disease progression in a previously characterized cohort of established CHC Egyptian patients. The second question is whether obesity, inflammation (TNF-*α*), and IR are cofounders for this association.

## 2. Subjects and Methods

### 2.1. Subjects

The studied groups included (85) subjects. Twelve subjects served as healthy controls (group I) selected from healthy subjects working in, or attending with their relatives, to the outpatient clinics of Ain Shams University Specialized Hospitals (ASUSH). None of the healthy controls took any medication or dietary supplements including vitamin(s) and/or antioxidant(s). 

 Seventy three patients with CHC were selected from Cairo Liver Center, Dokki, Cairo, Egypt. After protocol approval, the study was conducted in the period from December 2009 to December 2010. All subjects gave written informed consent prior to participation and the study was approved by the Committees on Medical Ethics of the Cairo Liver Center and ASUSH. The study was carried out in accordance with the regulations and recommendations of the Declaration of Helsinki. 

Patients enrolled in the study were classified into the following groups: asymptomatic chronic hepatitis C carriers (ASC) (group II) (*n* = 20) with normal liver function tests (LFTs), namely, alanine transaminase; ALT, for at least 6 months. Group III were patients with liver fibrosis (*n* = 20) and patients with cirrhosis (group IV) (*n* = 33). Histopathological staging was done using METAVIR scoring system where fibrosis was staged as (F1–F3) and (F4) represents established cirrhosis [21]. Cirrhosis was previously diagnosed by evidence Stigmata, lab, and radiographic procedures. Steatosis was assessed by liver biopsy and features detected by ultrasound. 

 The following exclusion criteria were used for all subjects: hypertension, cardiopulmonary disease, renal disease, endocrine disorders including diabetes, malignancy, having previous interferon treatment or recently received any antiinflammatory drugs, as well as smokers and alcoholics, and other causes of CLD as chronic hepatitis B, autoimmune hepatitis, acute hepatitis, haemochromatosis, hepatocellular carcinoma, and biliary disorders. Additional criteria for chronic hepatitis patients include age at diagnosis ≥40 years.

### 2.2. Methods

#### 2.2.1. Data Collection

A detailed drug treatment(s) history was collected, and physical examination of the patients was carried out with special emphasis on previous surgical procedures and prior parenteral therapy. Complete clinical examination was done, on the day of sample withdrawal which included the manifestations of hepatitis such as hepatomegaly, tenderness in the right hypochondrium, splenomegaly, and lower limb edema, or liver cell failure such as jaundice, hepatic encephalopathy, bleeding varices, and ascites. Biopsy samples were assessed unless contraindication or established cirrhosis was present; abdominal ultrasonography and endoscopy were also done side by side with routine laboratory investigations including complete blood picture, liver, and kidney function tests. HCV was diagnosed by anti-HCV assay by a third generation enzyme immunoassay, and HCV RNA by real time PCR. Medical records indicated that all patients were of HCV genotype 4 which is the most common in Egyptian patients.

Body mass index (BMI) was calculated as an index of the weight (in kilograms) divided by the square of the height (in meters) measured on the same day of sample withdrawal. Centers for Disease Control and Prevention (CDC) classify the normal range of BMI to be between 18.5–24.9 kg/m^2^, overweight BMI between 25–29.9 kg/m^2^, and the obese BMI > 30 kg/m^2^ [22]. Since BMI could be influenced by the presence of ascites in patients with cirrhosis, earlier weights taken prior to the diagnosis of ascites were used for the calculation of BMI [23].

#### 2.2.2. Sample Preparation, Collection, and Storage

All subjects were advised to take no medication on the morning before blood sample collection. Fasting blood (5 mL) was obtained from the antecubital vein, after an overnight fasting period (10–12 hours). Samples were divided into two parts; one containing trisodium citrate (final concentration 1 mg/mL) for prothrombin time (PT) determination. The other part was taken into vacutainer clotted tubes, where sera were separated by centrifugation at 3000 rpm for 10 min for other lab measurements. Other sera aliquots were kept frozen at −70°C for measurement of serum apelin (with no need for aprotinin pretreatment step applied to plasma samples [24]), TNF-*α*, and insulin.

#### 2.2.3. Laboratory Assessments

Sera were obtained, aliquoted for the measurement of LFTs: aspartate transaminase (AST), ALT, bilirubin, albumin, fasting blood glucose (FBG), and lipids (total cholesterol (TC) and triacylglycerol (TAG)) by using standard enzymatic techniques using appropriate kits and semiautomated photometer 5010. Determination of serum apelin (Phoenix Pharmaceuticals, Burlingame, Calif) using the apelin-12 microplate enzyme-linked immunosorbant assay (ELISA) kit, TNF-*α* (Orgenium laboratories ELISA), and insulin (Monobind Inc.) using enzyme-linked immunosorbant assay (ELISA) kits, and, following ELISA, procedures were carried out according to the manufacturers' instructions.

Insulin resistance was determined by the homeostasis model of assessment (HOMA) [25] using the formula: fasting insulin (*μ*IU/mL) × fasting blood glucose (mg/dL)/405.

### 2.3. Statistical Analysis

SPSS statistics (V. 19.0, IBM Corp., USA, 2010) was used for data analysis. Data was expressed as mean ± S.D for quantitative parametric measures, in addition to median and IQR for nonparametric data and percentiles for categorical data. Student's *t* test was used for comparison of two independent groups for parametric data and Wilcoxon Rank Sum for nonparametric data. However, for comparison between more than 2 patient groups for parametric data, we used analysis of variance (ANOVA). Multiple comparisons (Post hoc test: LSD (least significant difference)) were also followed to investigate the possible statistical significance between each 2 groups. Moreover, comparison between more than 2 patients' groups for nonparametric data Kruskall Wallis test was used. Finally, Spearman's ranked correlation test, to study the possible association between each two variables among each group for nonparametric data, using the probability of error at 0.05 was considered significant, while at 0.01 and 0.001 were highly significant. Multiple linear regression analysis was done on all measured parameters to allow for adjustment of apelin level.

## 3. Results

No significant differences with regards to age or gender distribution existed among groups. The clinical and demographic characteristics as well as studied parameters of all participants (control and CHC patients) are shown in Table 1. Significant differences in median values of apelin, TNF-*α*, and HOMA-IR levels existed between control and CHC patients as shown in Table 1. 

 CHC patients were subdivided into three groups: ASC (20 patients), fibrotic (20 patients), and cirrhotic (33 patients). Laboratory investigations and studied parameters for each group are detailed in Table 2. Regarding IR, it was significantly higher in all groups when compared to control. It was also significantly higher in cirrhotic patients (4.1) in comparison to ASC (2.75). TNF-*α* level was also higher in all groups in comparison to controls with median (76.6) being highest among ASC (616). 

 HCV-related disease progression was associated with greater median values of apelin compared to nondiseased control patients, while those with cirrhosis had the highest apelin concentrations among those with liver disease, with median value (3.9). Patients with fibrosis also displayed higher median values of apelin (3) when compared to either controls (1.11) or ASC (2), with nonsignificant differences among different fibrosis Metavir scores. These results were consistent when apelin was adjusted for BMI, TAG, and TC covariants, whereas elevation in apelin levels disappeared only in case of ASC after adjustment as depicted in Table 2. 

Table 3 illustrates apelin levels among different 2 patient subgroups, where cirrhotic patients (*n* = 33) were further subdivided into: (a) patients not suffering from complications including portal hypertension and/or ascites (13 subjects); (b) patients encountering complications as portal hypertension and/or ascites (20 patients). Upon comparing adjusted-apelin level among the two subgroups, it was found to be significantly higher in those with complications with median (2.36) in comparison to those free of such complications (1.87) as shown in (Table 3). 

 In an attempt to test our second aim reclaiming whether obesity is a cofounder for apelin serum levels association with CHC disease progression, we analyzed apelin levels in the obese (36 patients) versus lean (37 patients). It was found to be significantly higher in the latter with median value (3.6) versus (2.96) in the former (Table 4).

Additionally, CHC patients were subdivided into IR (43 patients) and non-IR (30 patients). Adjusted-apelin level was significantly higher in IR subjects with median (1.9) than non-IR ones (1.7) (Table 4). 

**Figure 1:**
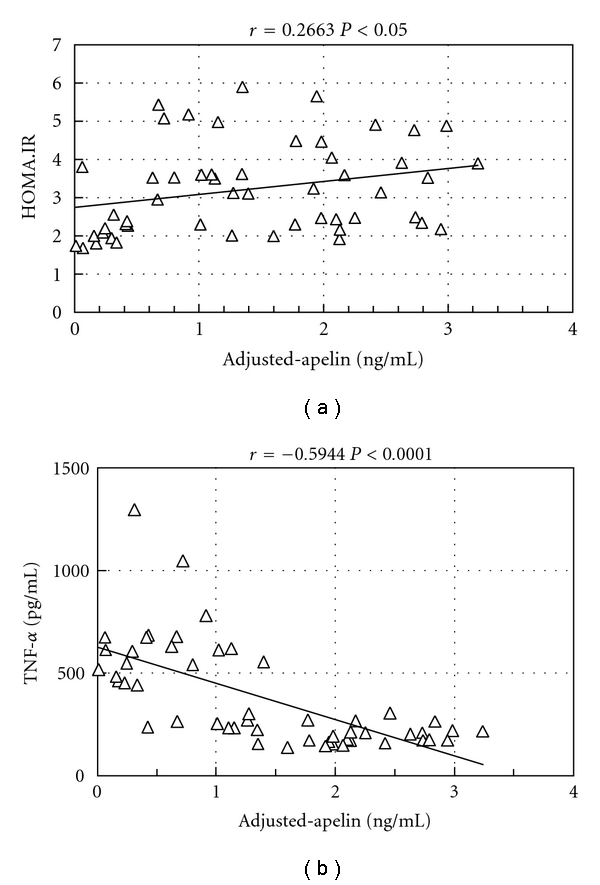
(a) Regression analysis showing correlation between adjusted-apelin and HOMA.IR among CHC patients (*n* = 73). (b) Regression analysis showing correlation between adjusted-apelin and TNF-*α* among CHC patients (*n* = 73).

Correlations of adjusted-apelin levels to different parameters showed modest positive relation to IR in all CHC patients (*r* = 0.2663, *P* < 0.05) (Figure 1(a)). Such relation appeared to be significant in group of ASC (*r* = 0.665, *P* < 0.01) but was nonsignificant for fibrotic and cirrhotic groups (Table 5).

Additionally, adjusted-apelin was found to be negatively correlated to TNF-*α* in all CHC patients (*n* = 73) with high significance (*r* = −0.5994, *P* < 0.0001) (Figure 1(b)). Further analysis within patients groups revealed significant negative correlation in the cirrhotic group (*r* = −0.488, *P* < 0.01) (Table 5). However, it was nonsignificant for both ASC and fibrotic groups.

## 4. Discussion 

Recent emerging studies pointed to the possible multiple effects of the apelinergic system in the liver and related it to oxidative stress, inflammation [8], fibrosis [10], angiogenesis [26], as well as haemodynamic and vascular disturbances [11, 12]. 

Apelin was evaluated in all groups both before and after adjustment for BMI, TAG, and TC that acted as potential covariants. Studies assessing apelin-36 and apelin-12 levels in patients with nonalcoholic fatty liver disease (NAFLD) indicated that the elevation of this peptide did not persist after adjustment for potential confounders and rather attributed apelin elevation in these cases to obesity and IR that are closely associated with NAFLD [27, 28]. 

In contrast to NAFLD models, the present study shows that serum apelin level is elevated in patients with either fibrosis or cirrhosis due to HCV, with significant differences among the two groups being higher in the latter, even after apelin adjustment. This is in line with previous investigations demonstrating that patients with cirrhosis showed significant increase in apelin circulating levels [11, 29]. 

Moreover, recent emerging studies speculated that activated hepatic stellate cells (HSCs) represent a potential source for apelin in liver [11] and that apelin could be an important mediator of the profibrogenic gene induction that markedly stimulates collagen-I synthesis [10]. In addition, apelin contributes to platelet-derived growth factor-induced proliferation of HSC's in vitro [12], all of which are known to contribute largely to fibrosis progression and extracellular matrix deposition [30, 31]. In this setting, we also found apelin serum level to be significantly elevated in patients with fibrosis. Thus, apelin emerges as a major contributor to the fibrogenic process(es) occurring in liver disease [10] as well as playing role in disease progression. 

On the other hand, this elevation in case of ASC disappeared after apelin adjustment to cofounders, which points to the interference of these cofactors in elevating apelin levels and may result in the early upset of the system in CHC patients. 

Moreover, the current study demonstrated that this peptide is closely associated to ascites and portal hypertension complications. Tiani and his coworkers suggested that the expression of endogenous apelin/APJ signaling is associated with development of portal hypertension and contributes to the formation of portosystemic collateral blood vessels and splanchnic neovascularization in portal hypertensive rats [26]. 

In the last few years, several data have accumulated suggesting that obesity also plays role in development and progression of liver disease of well-defined etiology [32]. Recently, expression of both apelin and APJ has been described in adipocytes [7] and is suggested to stimulate blood vessel growth, due to its proangiogenic activity, thus leading to increased growth of adipose tissue [33]. Coinciding with that line, our study also demonstrated that obese subjects with CHC had significant increased circulating apelin levels than lean patients, regardless of the stage or grade of liver disease.

 On the other hand, studies also demonstrated that apelin expression was higher in animal models of obesity associated with hyperinsulinemia [7], in addition to its role in adipogenesis [33] and steatosis [8]; all of which contribute largely to fibrosis progression, as well as higher degree of inflammation [32, 34]. Besides, virus C infection may induce IR by blocking intracellular signaling [35]. Further insight in our study revealed that IR was significantly higher among all groups of CHC in comparison to the control group. Moreover, significant difference was also found between ASC and cirrhotic groups, being higher in the latter. Moreover, when comparing apelin levels in IR and non-IR groups, it was significantly higher in IR group. Additionally, there was significant positive correlation between IR and adjusted-apelin in patients with CHC. This is in agreement with a previous study conducted by Aktas et al., who depicted that apelin, the novel adipokine, was associated with the components of the metabolic syndrome (hyperlipidemia, obesity, and IR) in NAFLD patients [27]. However, our correlation was quite significant in ASC but was unexpectedly nonsignificant in the case of progressive stages of fibrosis and cirrhosis, suggesting that the upset of the apelin system in these stages may follow a unique different pattern irrelevant to IR.

 Moreover, previous investigations had pointed to TNF-*α* as an inductor of apelin synthesis in adipocytes [36]. Hence, we sought to investigate such correlation in our CHC population. TNF-*α* was mostly elevated in cases of ASC, this was in agreement with Zekri and his coworkers [37] but was opposite to what was reported by Goyal et al. [38] and Toyoda et al. [39]. This could be attributed to the difference in the epitopes of the ELISA system used by the different investigating groups or to the difference in genotypes where all our patients were HCV genotype 4. Moreover, the striking elevation of this proinflammatory cytokine in carriers may reflect both insufficiencies of HCV elimination and/or a failure to control the cytokine cascade [37]. 

 In the current study, negative correlation was found between TNF-*α* and adjusted-apelin in patients with CHC. Further analysis of such correlation in CHC groups revealed a positive correlation between TNF-*α* and apelin in cases of ASC but was nonsignificant after adjustment for covariants, emphasizing the role of covariants in the induction of apelin in early stages of hepatitis. Interestingly, in case of, cirrhosis, there was significant negative correlation, suggesting that apelin induction in CLD follows alternative signaling pathways. This is in agreement with Melgar-Lesmes and his coworkers who pointed to the diminution of apelin expression induced by TNF-*α* in HSC cultures and explained that it could represent a homeostatic protective response toward reducing the overactivated hepatic apelin system in patients with advanced liver disease [10]. 

 These findings point to the possible role of apelin in CLD progression. Moreover, this provides a rationale to investigate new drugs targeting the apelin/APJ signaling pathway to reduce fibrosis and to improve hemodynamics in those patients. 

## 5. Conclusion

Circulating serum apelin level varies in different stages of CHC, which in conjunction with IR and obesity, would contribute to fibrosis progression. Additionally, apelin is more elevated in patients with ascites and portal hypertension and may have role in the development of these complications. Apelin, being recently added to the list of “adiposity signals,” in this setting, apelin targeting could be of additional benefit in obese patients with concomitant CLD, for alleviating both conditions and possibly resulting in better outcomes.

##  Conflict of Interests 

The authors declare that there is no Conflict of Interest.

## Figures and Tables

**Table 1:** Demographic, clinical characteristics, and studied parameters of all participants.

Groups/parameters	Control	Liver disease	*P*
*n*	12	73	
Age (years)	51 ± 3.7	52.4 ± 5.8	NS
Gender (M/F) Φ	6/6	48/25	NS
BMI (Kg/m^2^)	23 ± 1.6	28.7 ± 5.2	
BMI (18.5–24.9), lean *n* (%)	12 (100%)	37 (51%)	
BMI (30–35), obese *n* (%)	0	36 (49%)	
ASC/fibrosis/cirrhosis	—	20/20/33	
Fatty liver	—	14 (19%)	
Portal hypertension* and/or ascites	—	20 (27%)	
HOMA-IR^§^	1.85 (1.63–2)	3.28 (2.3–4.7)	0.001
IR *n* (%)	—	43 (59%)	
TNF-*α* (pg/mL)^§^	76.6 (43–141)	297.0 (205.5– 457.2)	<0.0001
Apelin (ng/mL)^§^	1.11 (0.83–1.42)	3.25 (2.6–3.9)	<0.0001

Data are mean *± *S.D, Φ-male to female ratio, § = median (25th and 75th centiles-quartiles). *surrogate markers of portal hypertension, esophageal varices, splenomegaly, and thrombocytopenia.

**Table 2:** Serum concentrations of the studied parameters in the different studied groups.

Groups, *n*/Parameter	Control, 12	Liver diseases, 73			*P*1	*P*2	*P*3
		ASC, 20	Fibrosis, 20	Cirrhosis, 33			
AST (U/L)	23.6 ± 2	36 ± 7.5	70 ± 10	69 ± 20	NS	<0.0001	<0.0001
ALT^§^ (U/L)	26 (23–28)	37.5 (28–50)	100 (40–140)	43 (34–60)	NS	0.015	NS
Bilirubin § (mg/dL)	0.7 (0.6–0.7)	0.75 (0.6–1)	0.9 (0.7–1.1)	2.2 (1.2–2.95)	NS	<0.0001	0.001
Albumin (gm/dL)	3.86 ± 0.27	4 ± 0.22	3.9 ± 0.32	2.8 ± 0.6	NS	0.009	<0.0001
PT %	85 ± 7.3	81 ± 7.3	73.3 ± 6.8	52 ± 13.4	0.019	<0.0001	<0.0001
Glucose (mg/dL)	82 ± 8.7	98 ± 8.5	87 ± 10.7	88 ± 10.7	0.001	NS	0.01
TC (mg/dL)	167 ± 21	176 ± 11.6	177.5 ± 16.2	182 ± 16	NS	NS	NS
TAG (mg/dL)	100 ± 21	123 ± 37	134 ± 33	122 ± 25	NS	NS	NS
HOMA-IR^§^	1.85 (1.63–2)	2.75 (1.9–3.6)^a^	4.26 (2.4–5.8)^a^	4.1 (2.42–7.7)^a^	NS	NS	0.015
IR *n* (%)	0	8 (40%)	12 (60%)	23 (70%)			
TNF-*α* (pg/mL)^§^	76.6 (43–141)	616 (535–694)^a^	205 (157–267)^a^	280 (205–337)^a^	0.014	0.002	0.004
Apelin (ng/mL)^§^	1.11 (0.83–1.42)	2 (1–2.54)^a^	3 (2.9–3.3)^a^	3.9 (3.6–4)^a^	0.001	<0.0001	<0.0001
Apelin-adj (ng/mL)^§^	0.8 (0.3–1)	0.52 (0.16–0.93)	1.77 (1.17–2)^a^	2.3 (1.9–2.6)^a^	0.005	<0.0001	<0.0001

Data are mean *± *S.D, § = median (25th and 75th centiles-quartiles).  ^a^significant difference from healthy controls at *P* ≤ 0.05. AST: Aspartate transaminase; ALT: Alanine Transaminase; PT: Prothrombin time; TC: total cholesterol; TAG: triacylglycerols. *P*1 for comparison of ASC and fibrosis groups, *P*2 for comparison of fibrosis and cirrhosis groups, and *P*3 for comparison of ASC and cirrhosis groups.

**Table 3:** Adjusted-apelin levels among cirrhotic patients group (*n* = 33).

Cirrhotic patients group	*n*	Adjusted-apelin (ng/mL)
Free of complications	13	1.87 (1.33–2.4)
With complications	20	2.36 (1.55–2.6)^a^

Data are medians (25th and 75th centiles-quartiles).   ^a^significant difference from cirrhotic patients free of complications (PH and/or ascites) at *P* ≤ 0.05.

**Table 4:** Apelin and adjusted-apelin levels among different CHC groups (*n* = 73).

	*n*	Apelin (ng/mL)	Adjusted-apelin (ng/mL)
Lean patients	37	2.96 (2.08–3.7)	
Obese patients	36	3.60 (2.95–3.9)^a^	

Non-IR patients	30		1.7 (0.82–2.2)
IR patients	43		1.9 (1.12–2.4)^b^

Data are medians (25th and 75th centiles-quartiles).   ^a^significant difference from lean patients at *P* ≤ 0.05.   ^b^significant difference from non-IR patients at *P* ≤ 0.05.

**Table 5:** Correlation coefficients (*r*) of different parameters in patients with CHC (*n* = 73).

Parameters/groups	Adjusted-apelin (ng/mL)		
	ASC	Fibrosis	Cirrhosis

*n*	20	20	33
TNF-*α* (pg/mL)	0.26^NS^	−0.212^NS^	−0.488**
HOMA-IR	0.665**	0.202^NS^	0.18^NS^

*r* = Spearman's rank correlation coefficients. **, ***Correlation is significant at the 0.01, 0.001 level. NS: nonsignificant correlation.
